# A-type Ce_2_NCl_3_

**DOI:** 10.1107/S1600536811023105

**Published:** 2011-06-18

**Authors:** Christian M. Schurz, Thomas Schleid

**Affiliations:** a Institut für Anorganische Chemie, Universität Stuttgart, Pfaffenwaldring 55, 70569 Stuttgart, Germany

## Abstract

Cerium(III) nitride chloride, Ce_2_NCl_3_, contains *trans*-edge connected [NCe_4_]^9+^ tetra­hedra (222 symmetry) forming chains parallel to the *c* axis that are separated by Cl^−^ anions. The Ce^3+^ cations (..*m* symmetry) are each surrounded by two N^3−^ and six Cl^−^ anions in a bicapped trigonal prismatic coordination geometry (CN = 8).

## Related literature

For isotypic A-type *M*_2_NCl_3_ structures, see: Schurz & Schleid (2009 ▶) for La; Uhrlandt & Meyer (1995 ▶) for Pr. For a comparison of A-, B-, and C-type structures, see: Schurz & Schleid (2009 ▶); Schurz (2011 ▶).

## Experimental

### 

#### Crystal data

Ce_2_NCl_3_
*M* *_r_* = 400.60
Orthorhombic, 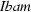

*a* = 13.6021 (9) Å
*b* = 6.8903 (5) Å
*c* = 6.1396 (4) Å
*V* = 575.42 (7) Å^3^
*Z* = 4
Mo *K*α radiation
μ = 16.86 mm^−1^
*T* = 293 K
0.19 × 0.14 × 0.10 mm

#### Data collection

Nonius KappaCCD diffractometer
Absorption correction: numerical (*X-SHAPE*; Stoe & Cie, 1999 ▶) *T*_min_ = 0.085, *T*_max_ = 0.198
4016 measured reflections
385 independent reflections
329 reflections with *I* > 2σ(*I*)
*R*_int_ = 0.082

#### Refinement

*R*[*F* ^2^ > 2σ(*F* ^2^)] = 0.035
*wR*(*F* ^2^) = 0.061
*S* = 1.12
385 reflections
20 parameters
Δρ_max_ = 1.06 e Å^−3^
Δρ_min_ = −1.09 e Å^−3^

### 

Data collection: *COLLECT* (Nonius, 1998 ▶); cell refinement: *SCALEPACK* (Otwinowski & Minor, 1997 ▶); data reduction: *SCALEPACK* and *DENZO* (Otwinowski & Minor, 1997 ▶); program(s) used to solve structure: *SHELXS97* (Sheldrick, 2008 ▶); program(s) used to refine structure: *SHELXL97* (Sheldrick, 2008 ▶); molecular graphics: *DIAMOND* (Brandenburg, 2006 ▶); software used to prepare material for publication: *SHELXL97*.

## Supplementary Material

Crystal structure: contains datablock(s) I, global. DOI: 10.1107/S1600536811023105/mg2121sup1.cif

Structure factors: contains datablock(s) I. DOI: 10.1107/S1600536811023105/mg2121Isup2.hkl

Additional supplementary materials:  crystallographic information; 3D view; checkCIF report

## Figures and Tables

**Table 1:** Selected bond lengths (Å)

Ce—N^i^	2.3414 (4)
Ce—N	2.3414 (4)
Ce—Cl2^ii^	2.873 (3)
Ce—Cl2	2.969 (3)
Ce—Cl1	2.9876 (5)
Ce—Cl1^iii^	2.9876 (5)
Ce—Cl2^iv^	3.3896 (11)
Ce—Cl2^v^	3.3896 (11)

Symmetry codes: (i) 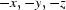; (ii) 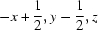; (iii) 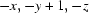; (iv) 
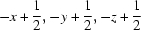
; (v) 
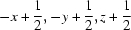
.

**Fig. 1.**
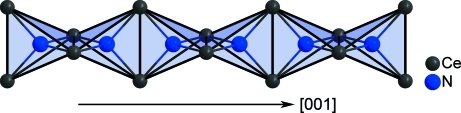
Chains of trans-edge connected [NCe4]9+ tetrahedra in A-type Ce2NCl3.

**Fig. 2.**
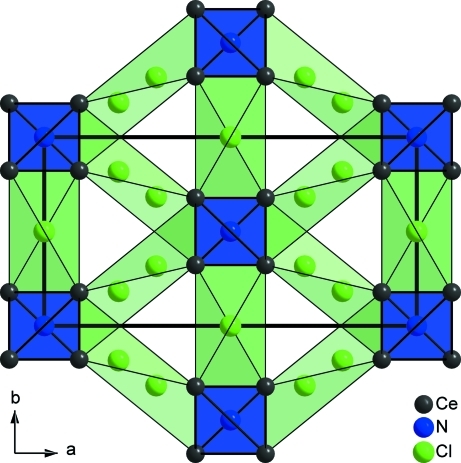
Polyhedral representation of A-type Ce2NCl3.

**Fig. 3.**
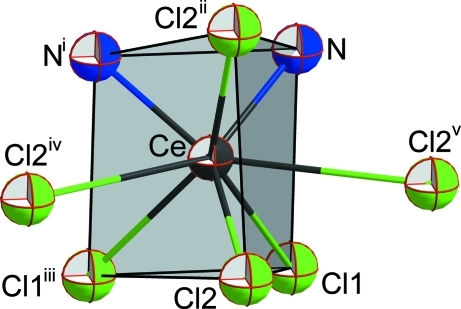
Coordination sphere of Ce atoms in A-type Ce2NCl3. Displacement ellipsoids are drawn at 90% probability level. Symmetry codes: (i) -x, -y, -z; (ii) -x + 1/2, y - 1/2, z; (iii) -x, -y + 1, -z; (iv) -x + 1/2, -y + 1/2, -z + 1/2; (v) -x + 1/2, -y + 1/2, z + 1/2.

## supplementary crystallographic information

### Comment

Lanthanide nitride chlorides *M*_2_NCl_3_ adopt A-, B-, or C-type structures (Schurz & Schleid, 2009; Schurz, 2011). Ce_2_NCl_3_ belongs to the short series of orthorhombic A-type structures formed for *M* = La–Pr (Uhrlandt & Meyer, 1995; Schurz & Schleid, 2009). The structure features *trans*-edge connected [NCe_4_]^9+^ tetrahedra (222 symmetry) forming straight infinite chains running parallel to the *c*-axis (Fig. 1). These chains are bundled in a hexagonal arrangement and are interconnected by (Cl1)^–^ anions (222 symmetry) along [110] and [110], and by (Cl2)^–^ anions (..*m* symmetry) along [010] (Fig. 2). Both types of chloride anions show a fourfold surrounding of cerium cations, while the coordination geometry of the trivalent cerium cations (..*m* symmetry) can be described as bicapped trigonal prismatic (CN = 8) with two Ce–N and six Ce–Cl contacts (Fig. 3).

### Experimental

Light yellow, transparent, needle-shaped crystals of Ce_2_NCl_3_ were obtained as the main product after a mixture of 0.06 g Ce, 0.13 g CeCl_3_, and 0.01 g NaN_3_, along with 0.30 g NaCl added as a flux, was heated at 850 °C for 7 days in a sealed, evacuated fused-silica vessel.

### Crystal data

**Table:**

Ce_2_NCl_3_	*F*(000) = 696
*M**_r_* = 400.60	*D*_x_ = 4.624 Mg m^−^^3^
Orthorhombic, *I**b**a**m*	Mo *K*α radiation, λ = 0.71069 Å
Hall symbol: -I 2 2c	Cell parameters from 19363 reflections
*a* = 13.6021 (9) Å	θ = 0.4–28.3°
*b* = 6.8903 (5) Å	µ = 16.86 mm^−^^1^
*c* = 6.1396 (4) Å	*T* = 293 K
*V* = 575.42 (7) Å^3^	Needle, light yellow
*Z* = 4	0.19 × 0.14 × 0.10 mm

### Data collection

**Table:**

Nonius KappaCCD diffractometer	385 independent reflections
Radiation source: fine-focus sealed tube	329 reflections with *I* > 2σ(*I*)
graphite	*R*_int_ = 0.082
ω and φ scans	θ_max_ = 28.1°, θ_min_ = 3.0°
Absorption correction: numerical (*X-SHAPE*; Stoe & Cie, 1999)	*h* = −18→18
*T*_min_ = 0.085, *T*_max_ = 0.198	*k* = −9→9
4016 measured reflections	*l* = −8→8

### Refinement

**Table:**

Refinement on *F*^2^	Primary atom site location: structure-invariant direct methods
Least-squares matrix: full	Secondary atom site location: difference Fourier map
*R*[*F*^2^ > 2σ(*F*^2^)] = 0.035	*w* = 1/[σ^2^(*F*_o_^2^) + (0.0169*P*)^2^ + 3.8817*P*] where *P* = (*F*_o_^2^ + 2*F*_c_^2^)/3
*wR*(*F*^2^) = 0.061	(Δ/σ)_max_ < 0.001
*S* = 1.12	Δρ_max_ = 1.06 e Å^−^^3^
385 reflections	Δρ_min_ = −1.09 e Å^−^^3^
20 parameters	Extinction correction: *SHELXL97* (Sheldrick, 2008), Fc^*^=kFc[1+0.001xFc^2^λ^3^/sin(2θ)]^-1/4^
0 restraints	Extinction coefficient: 0.0006 (3)

### Special details

**Table:**

Geometry. All e.s.d.'s (except the e.s.d. in the dihedral angle between two l.s. planes) are estimated using the full covariance matrix. The cell e.s.d.'s are taken into account individually in the estimation of e.s.d.'s in distances, angles and torsion angles; correlations between e.s.d.'s in cell parameters are only used when they are defined by crystal symmetry. An approximate (isotropic) treatment of cell e.s.d.'s is used for estimating e.s.d.'s involving l.s. planes.
Refinement. Refinement of *F*^2^ against ALL reflections. The weighted *R*-factor *wR* and goodness of fit *S* are based on *F*^2^, conventional *R*-factors *R* are based on *F*, with *F* set to zero for negative *F*^2^. The threshold expression of *F*^2^ > σ(*F*^2^) is used only for calculating *R*-factors(gt) *etc*. and is not relevant to the choice of reflections for refinement. *R*-factors based on *F*^2^ are statistically about twice as large as those based on *F*, and *R*- factors based on ALL data will be even larger.

### Fractional atomic coordinates and isotropic or equivalent isotropic displacement parameters (Å^2^)

**Table:**

	*x*	*y*	*z*	*U*_iso_*/*U*_eq_	
Ce	0.09389 (4)	0.17747 (9)	0.0000	0.0243 (3)	
N	0.0000	0.0000	0.2500	0.028 (3)	
Cl1	0.0000	0.5000	0.2500	0.0330 (8)	
Cl2	0.30050 (19)	0.3163 (4)	0.0000	0.0390 (7)	

### Atomic displacement parameters (Å^2^)

**Table:**

	*U*^11^	*U*^22^	*U*^33^	*U*^12^	*U*^13^	*U*^23^
Ce	0.0238 (3)	0.0259 (4)	0.0231 (3)	−0.0019 (3)	0.000	0.000
N	0.026 (6)	0.038 (8)	0.022 (6)	0.000	0.000	0.000
Cl1	0.056 (2)	0.0229 (19)	0.0199 (15)	0.000	0.000	0.000
Cl2	0.0319 (14)	0.0361 (15)	0.0489 (15)	−0.0092 (12)	0.000	0.000

### Geometric parameters (Å, °)

**Table:**

Ce—N^i^	2.3414 (4)	Ce—Ce^viii^	3.9934 (7)
Ce—N	2.3414 (4)	Ce—Ce^ix^	3.9934 (7)
Ce—Cl2^ii^	2.873 (3)	N—Ce^i^	2.3414 (4)
Ce—Cl2	2.969 (3)	N—Ce^ix^	2.3414 (4)
Ce—Cl1	2.9876 (5)	N—Ce^vii^	2.3414 (4)
Ce—Cl1^iii^	2.9876 (5)	Cl1—Ce^x^	2.9876 (5)
Ce—Cl2^iv^	3.3896 (11)	Cl1—Ce^ix^	2.9876 (5)
Ce—Cl2^v^	3.3896 (11)	Cl1—Ce^iii^	2.9876 (5)
Ce—Ce^i^	3.5362 (12)	Cl2—Ce^xi^	2.873 (3)
Ce—Ce^vi^	3.9249 (8)	Cl2—Ce	2.969 (3)
Ce—Ce^vii^	3.9249 (8)	Cl2—Ce^v^	3.3896 (11)
			
N^i^—Ce—N	81.923 (19)	Ce^i^—Ce—Ce^vii^	64.473 (17)
N^i^—Ce—Cl2^ii^	79.66 (4)	Ce^vi^—Ce—Ce^vii^	102.91 (3)
N—Ce—Cl2^ii^	79.66 (4)	N^i^—Ce—Ce^viii^	31.484 (13)
N^i^—Ce—Cl2	133.21 (3)	N—Ce—Ce^viii^	98.92 (2)
N—Ce—Cl2	133.21 (3)	Cl2^ii^—Ce—Ce^viii^	108.65 (3)
Cl2^ii^—Ce—Cl2	78.80 (4)	Cl2—Ce—Ce^viii^	127.261 (18)
N^i^—Ce—Cl1	119.48 (2)	Cl1—Ce—Ce^viii^	96.980 (16)
N—Ce—Cl1	79.545 (12)	Cl1^iii^—Ce—Ce^viii^	48.061 (9)
Cl2^ii^—Ce—Cl1	149.085 (6)	Ce^i^—Ce—Ce^viii^	62.486 (16)
Cl2—Ce—Cl1	99.49 (5)	Ce^vi^—Ce—Ce^viii^	53.041 (12)
N^i^—Ce—Cl1^iii^	79.545 (12)	Ce^vii^—Ce—Ce^viii^	126.959 (11)
N—Ce—Cl1^iii^	119.48 (2)	N^i^—Ce—Ce^ix^	98.92 (2)
Cl2^ii^—Ce—Cl1^iii^	149.085 (6)	N—Ce—Ce^ix^	31.484 (13)
Cl2—Ce—Cl1^iii^	99.49 (5)	Cl2^ii^—Ce—Ce^ix^	108.65 (3)
Cl1—Ce—Cl1^iii^	61.829 (12)	Cl2—Ce—Ce^ix^	127.261 (18)
N^i^—Ce—Ce^i^	40.961 (9)	Cl1—Ce—Ce^ix^	48.061 (9)
N—Ce—Ce^i^	40.961 (9)	Cl1^iii^—Ce—Ce^ix^	96.980 (16)
Cl2^ii^—Ce—Ce^i^	76.24 (6)	Ce^i^—Ce—Ce^ix^	62.486 (15)
Cl2—Ce—Ce^i^	155.05 (6)	Ce^vi^—Ce—Ce^ix^	126.959 (11)
Cl1—Ce—Ce^i^	101.87 (2)	Ce^vii^—Ce—Ce^ix^	53.041 (11)
Cl1^iii^—Ce—Ce^i^	101.87 (2)	Ce^viii^—Ce—Ce^ix^	100.48 (2)
N^i^—Ce—Ce^vi^	33.054 (12)	Ce^i^—N—Ce^ix^	113.89 (2)
N—Ce—Ce^vi^	100.80 (2)	Ce^i^—N—Ce^vii^	117.03 (3)
Cl2^ii^—Ce—Ce^vi^	57.34 (2)	Ce^ix^—N—Ce^vii^	98.077 (19)
Cl2—Ce—Ce^vi^	101.59 (3)	Ce^i^—N—Ce	98.077 (19)
Cl1—Ce—Ce^vi^	149.920 (11)	Ce^ix^—N—Ce	117.03 (3)
Cl1^iii^—Ce—Ce^vi^	93.536 (8)	Ce^vii^—N—Ce	113.89 (2)
Ce^i^—Ce—Ce^vi^	64.473 (17)	Ce^x^—Cl1—Ce^ix^	118.171 (12)
N^i^—Ce—Ce^vii^	100.80 (2)	Ce^x^—Cl1—Ce^iii^	83.877 (19)
N—Ce—Ce^vii^	33.054 (12)	Ce^ix^—Cl1—Ce^iii^	129.39 (2)
Cl2^ii^—Ce—Ce^vii^	57.34 (2)	Ce^x^—Cl1—Ce	129.39 (2)
Cl2—Ce—Ce^vii^	101.59 (3)	Ce^ix^—Cl1—Ce	83.877 (19)
Cl1—Ce—Ce^vii^	93.536 (8)	Ce^iii^—Cl1—Ce	118.171 (13)
Cl1^iii^—Ce—Ce^vii^	149.920 (11)	Ce^xi^—Cl2—Ce	138.81 (11)

Symmetry codes: (i) −*x*, −*y*, −*z*; (ii) −*x*+1/2, *y*−1/2, *z*; (iii) −*x*, −*y*+1, −*z*; (iv) −*x*+1/2, −*y*+1/2, −*z*+1/2; (v) −*x*+1/2, −*y*+1/2, *z*+1/2; (vi) *x*, −*y*, −*z*−1/2; (vii) *x*, −*y*, −*z*+1/2; (viii) −*x*, *y*, *z*−1/2; (ix) −*x*, *y*, *z*+1/2; (x) *x*, −*y*+1, −*z*+1/2; (xi) −*x*+1/2, *y*+1/2, *z*.
